# The Oral, Gut Microbiota and Cardiometabolic Health of Indigenous Orang Asli Communities

**DOI:** 10.3389/fcimb.2022.812345

**Published:** 2022-04-22

**Authors:** Li-Fang Yeo, Soo Ching Lee, Uma Devi Palanisamy, BAK. Khalid, Qasim Ayub, Shu Yong Lim, Yvonne AL. Lim, Maude Elvira Phipps

**Affiliations:** ^1^ Jeffrey Cheah School of Medicine & Health Sciences, Monash University Malaysia, Bandar Sunway, Malaysia; ^2^ Tropical Medicine and Biology Multidisciplinary Platform, School of Science, Monash University Malaysia, Bandar Sunway, Malaysia; ^3^ Department of Parasitology, Faculty of Medicine, Universiti Malaya, Kuala Lumpur, Malaysia; ^4^ Monash University Malaysia Genomics Facility, Monash University Malaysia, Bandar Sunway, Malaysia

**Keywords:** gut microbiota, oral microbiota, cardiometabolic health, orang asli, indigenous health

## Abstract

The Orang Asli (OA) of Malaysia have been relatively understudied where little is known about their oral and gut microbiomes. As human health is closely intertwined with the human microbiome, this study first assessed the cardiometabolic health in four OA communities ranging from urban, rural to semi-nomadic hunter-gatherers. The urban Temuan suffered from poorer cardiometabolic health while rural OA communities were undergoing epidemiological transition. The oral microbiota of the OA were characterised by sequencing the V4 region of the 16S rRNA gene. The OA oral microbiota were unexpectedly homogenous, with comparably low alpha diversity across all four communities. The rural Jehai and Temiar PP oral microbiota were enriched for uncharacterised bacteria, exhibiting potential for discoveries. This finding also highlights the importance of including under-represented populations in large cohort studies. The Temuan oral microbiota were also elevated in opportunistic pathogens such as *Corynebacterium, Prevotella*, and *Mogibacterium*, suggesting possible oral dysbiosis in these urban settlers. The semi-nomadic Jehai gut microbiota had the highest alpha diversity, while urban Temuan exhibited the lowest. Rural OA gut microbiota were distinct from urban-like microbiota and were elevated in bacteria genera such as *Prevotella 2, Prevotella 9*, Lachnospiraceae ND3007, and *Solobacterium.* Urban Temuan microbiota were enriched in *Odoribacter, Blautia, Parabacetroides, Bacteroides* and Ruminococcacecae UCG-013. This study brings to light the current health trend of these indigenous people who have minimal access to healthcare and lays the groundwork for future, in-depth studies in these populations.

## 1 Introduction

Improvement in sequencing technology, coupled with decreased costs and user-friendly, freely accessible bioinformatics tools, has increased the accessibility of microbiome research. Global microbiome studies have demonstrated that the human microbiome is complex and dynamic. Several large-scale, international, multi-ethnic microbiome projects have recently been launched (Hadrich, 2020). Yet, these studies still mainly involve WEIRD populations (Western, Educated, Industrialized, Rich, Democratic). A few Asian populations are relatively well studied, including China (Winglee et al., 2017; Zhang et al., 2019), Japan (Park et al., 2021), and Korea (Lim et al., 2021). However, there are many populations in Southeast Asia, especially the indigenous populations, where little to almost nothing is known about their microbiomes. Amongst them are the indigenous people of Peninsular Malaysia, also known as the Orang Asli (OA).

The OA are descendants of the early settlers who left Africa and arrived on the Malay Peninsular approximately 70,000 years ago (Hill et al., 2006). The OA are a minority, comprising just 0.7% of Malaysia’s total population (IWGIA, 2020). They are categorised into a total of 18 sub-groups or tribes and their communities are scattered across the peninsula (IWGIA, 2020). Some are urbanised and live near large cities. Some are semi-urbanised and live in resettlement villages, usually bordering the forest or palm oil plantations. A few small groups of OA still live in the interior rainforest where they lead semi-nomadic lifestyles and continue to practice hunting and gathering.

The OA lead unique lifestyles where their health and well-being are connected to the land that they live on. Urbanisation and land development have adversely affected their health. Many OA communities are undergoing an epidemiological transition. The disease burden is shifting from communicable diseases such as tuberculosis, leprosy, helminthiasis and malaria to non-communicable diseases such as cardiovascular diseases, type 2 diabetes, and obesity (Lim et al., 2009; Bedford, 2013; Phipps et al., 2015; Aghakhanian et al., 2019).

The link between the human microbiota and health has received much attention, especially in the field of personalized medicine and manipulation of the microbiota for therapeutics. The association between the human microbiota and cardiometabolic health is gaining attention (Fei et al., 2019). However, the association is not straightforward because cardiometabolic health includes a spectrum of health conditions which progress to metabolic syndrome (MetS) that eventually increases the risk of cardiovascular diseases (Vincent et al., 2017). There remains a dearth of information regarding OA cardiometabolic health. Dirt road tracks, river tributaries, and lakes limit accessibility to OA villages, which in turn limits their access to health care facilities and researchers.

This study engaged three OA subgroups, namely Temuan, Temiar and Jehai. The degree of urbanisation of the OA communities varied from urban and rural to semi-nomadic hunter-gatherers living in the forest. Their current cardiometabolic health were assessed, followed by characterization of their oral and gut microbiota using targeted 16S rRNA gene sequencing.

## 2 Methodology

### 2.1 Ethics Statement

This investigation was approved by the Ministry of Health Malaysia (MOH) under National Medical Research Registry, MNDR ID #09-23-3913, JAKOA and Monash University Human Research Ethics Committee (MUHREC – ID #11794).

### 2.2 Study Site and Participants

We recruited a total of 216 participants from three subtribes change to subgroups from 2017-2019. The urban OA community was represented by Temuan (n=36) from Bukit Lanjan, Selangor. The semi-urban community was represented by Temiar participants (n=29), recruited from resettlement villages in Gua Musang, Kelantan (Temiar GM). Their village was 30 mins away from the nearest town and was surrounded by a higher frequency of land development and deforestation. We also used an archival dataset for rural Temiar (n=68) recruited in an earlier study (Temiar PP). They lived in Pos Piah village, a 90-min drive on dirt track from the nearest town in Perak (Yeo et al., 2019). The difference in distance of an indigenous habitation to the nearest town may affect accessibility to store-bought food and account for plausible differences in diet. Thus, Temiar PP and Temiar GM were analyzed as separate demographic groups. The semi-nomadic hunter-gatherers were the Jehai (n=83) from Royal Belum Rainforest, Perak.

The urban Temuan consumed a diet that closely resembled urban Malaysians such as rice, biscuit, bread, fish and chicken. They also had easy access to fast-food outlets that offered relatively cheaper alternatives. The Temuan appeared to consume a diet deficient in plant fibre. The semi-urban Temiar consumed a mixture of store-bought food, including rice, biscuit, canned food, bread, chicken, fish, and plants that they grew such as sweet potato leaves. Some reported occasional hunting of small mammals although those were exceedingly rare. The hunter-gather Jehai reported a diet that mostly consisted of various leafy greens that grew wild in the jungle, along with fishing. Hunting was reportedly less frequent among the Jehai.

### 2.3 Recruitment and Sample Collection

Informed consent from tribal leaders and elders were obtained before fieldwork commenced. Researchers returned to the village on a pre-agreed time and date for recruitment and sample collection. Participants over 18 years old with no visible health ailments, who could communicate in Malay and provide informed consent were recruited. Participants who were pregnant, had a history of alcohol/drug abuse, or with known chronic illness (i.e. kidney failure, cancer, heart diseases) were excluded. Interviews were conducted in Malay to document the participant’s smoking habits, chewing of betel-quid nuts, and a descriptive dietary recall. Raw saliva and stool samples were collected without preservatives and stored on dry ice before being brought back to Monash University Malaysia.

### 2.4 Cardiometabolic Measurements

Anthropometrics, including height, weight, waist circumference, and blood pressure, were measured according to previous protocols (Phipps et al., 2015; Yeo et al., 2019). HbA1c and blood lipid levels (Total Cholesterol – TC, Triglyceride -TG, High Density Lipoprotein – HDL, Low-Density Lipoprotein – LDL) were measured using a finger-prick method. TG/HDL was used as a surrogate biomarker for insulin resistance with a cut-off point of 0.9-1.7 (Mostafa et al., 2012). Lipoprotein ratios, TC/HDL and LDL/HDL were used as cardiometabolic risk markers (Millán et al., 2009)where they have been well utilized in Asian cohorts such as Japanese (Katakami et al., 2011) and Chinese (Wen et al., 2017). HbA1c level <5.7% was considered normal, 5.7-6.3% indicated pre-diabetes, while >6.3% indicated diabetes (WHO, 2011). Metabolic syndrome (MetS) was assessed if three or more of the following criteria were met, modified from Grundy et al. (2005) when appropriate:

i) Asian Waist circumference >90 (men); >80 in (women) (WHO, 2011);
ii) Blood pressure > 130/85 mmHg;
iii) TG level > 8.3 mmol/L;
iv) HDL level < 2.2 mmol/L (men); <2.8 mmol/L (women);
v) HbA1c >5.7% (Park et al., 2012).

Framingham Risk Score (FRS) was calculated to estimate cardiovascular disease risk for the next five years using a recalibrated FRS calculator validated in an Australian indigenous cohort (Hua et al., 2017).

### 2.5 DNA Extraction

Saliva and stool samples from Temuan and Temiar GM were extracted and sequenced at the V4 region (515F:GTGCCAGCMGCCGCGGTAA and 806R: GGACTACHVGGGTWTCTAAT) by uBiome Inc (Almonacid et al., 2017) as part of a sequencing grant before they ceased operations. Subsequently, saliva DNA from Temiar PP and Jehai were extracted in-house using a modified salting-out method (Quinque et al., 2006; Yeo et al., 2019). Stool DNA was extracted according to IHMS protocol Q using Qiagen QIAamp DNA mini kit (Costea et al., 2017). The V3-V4 region of the 16S rRNA gene was amplified using the following primers:

F: TCGTCGGCAGCGTCAGATGTGTATAAGAGACAGCCTACGGGNGGCWGCAG
R:GTCTCGTGGGCTCGGAGATGTGTATAAGAGACAGGACTACHVGGGTATCTAATCC (Klindworth et al., 2013).

The amplicons were then sequenced on Illumina MiSeq instrument using 2x250bp paired end reads

### 2.6 Microbiota Control

Saliva and stool kit negative controls were ultrapure water that underwent the same extraction and PCR amplification process as the biological samples. A mock community (ATCC MSA-1002) was used as a positive control.

### 2.7 Microbiota Analysis

The V4 reads were trimmed to 125bp by uBiome. Only the forward reads were used as the reads were too short to be merged. The V3-V4 datasets were trimmed to V4 region using CutAdapt (Martin, 2011) before the paired-ends were stitched using USEARCH v11.0667 (Edgar, 2010). The merged reads were truncated to 125bp to match the V4 dataset.

Raw reads were imported to QIIME2 – version 2021.4 (Estaki et al., 2020) and denoised on DADA2 (Callahan et al., 2016). An open-reference phylogenetic tree was built using SATé-enabled phylogenetic placement (SEPP) method (Janssen et al., 2018). Bacteria taxonomy was classified with a trained Naïve Bayes Classifier using Silva reference database version 12_8. Files were exported and analysed on R 4.0.3 (R Core Team, 2020).

Alpha diversity was calculated using microbial richness, Shannon Index (evenness) and Pielou’s evenness (richness + evenness). Core microbiota genera were identified at 90% sample prevalence at 0.1% detection threshold (relative abundance). Beta-diversity was calculated using centred-log-ratio (clr) transformed Euclidean distance metrics and plotted on PCoA and supervised CAP ordination plots. Multivariate PERMANOVA (Anderson, 2017) on adonis was conducted using clr-transformed Euclidean distance with BMI and age groups as covariates. PERMDISP was used to measure the homogeneity of sample dispersion. Univariate differential abundance analysis was performed using ALDEX2 (Fernandes et al., 2014). Differentially abundant taxa were determined as statistically significant taxa with Benjamini-Hochberg corrected p-value <0.01 and effect size > 1 or < -1.

## 3 Results

### 3.1 Urbanized OA had Poorer Cardiometabolic Health Compared to Rural, Semi Nomadic Hunter-Gatherers

Cardiometabolic health was assessed in 214 OA individuals with 134 women with a mean age of 33 ± 1.9 and 80 men with a mean age of 40 ± 3.4. General obesity was found in 30.37% of the OA (**Table 1**). The urban Temuan who live in the city had the highest prevalence of obesity at 70.59%, whereas the semi-nomadic Jehai who live in the forest had the lowest prevalence at 25.30%. More than half (58%) of the OA had increased waist circumference, indicating they were at risk for poorer health. Type 2 Diabetes (T2D), as indicated by HbA1c, was generally low among the OA at 6.9%. However, half of the Temiar participants in our study turned out to be pre-diabetic. Using TG/HDL as a surrogate marker for insulin resistance, our study revealed 25.4% of the OA to be at risk for insulin resistance.

**Table 1:** Anthropometrics and cardiometabolic health measures of OA.

	Category	Asian Criteria	Total (%, n = 214)	Male (%, n = 80)	Female (%, n = 134)	Jehai (Semi-Nomadic, n = 83) (%)	Temiar (Sub-Urban, n = 97) (%)	Temuan (Urban, n = 34) (%)
**BMI**	Underweight	<18.5	11.21	16.25	8.21	15.66	8.25	8.82
	Normal	18.5-22.9	32.71	35.00	31.34	42.17	28.87	20.59
	Overweight	23-29.9	25.70	26.25	25.37	16.87	42.27	0.00
	Obese	≥30	30.37	22.50	35.07	25.30	20.62	70.59
**Waist CM**	Healthy	<90cm (M), <80cm (F)	41.98	64.56	28.57	30.86	49.48	47.06
	Risk	>90cm (M),	58.02	35.44	71.43	69.14	50.52	52.94
		>80cm (F)						
**HbA1c (%)**	Normal	4.0-5.6	64.37	53.73	71.03	87.80	43.24	
	Pre-diabetes	5.7-6.4	28.74	40.30	21.50	7.32	50.00	
	Diabetes	≥6.5	6.90	5.97	7.48	4.88	6.76	
**IR (TG/HDL)**	Healthy		74.60	71.88	84.97	71.60	78.13	
	Insulin-resistant	0.9-1.7	25.40	28.13	15.03	28.40	21.88	
**Blood Pressure**	Low	<90/60 mmHg	1.40	0.00	2.22	0.00	3.06	0.00
	Normal	<120/80 mmHg	35.98	27.85	40.74	42.17	31.63	33.33
	Pre-hypertension	<130/80 mmHg	41.12	41.77	40.74	39.76	44.90	33.33
	Stage 1	<140/90 mmHg	14.49	21.52	10.37	9.64	15.31	24.24
	Stage 2	<180/90 mmHg	6.07	7.59	5.19	7.23	4.08	9.09
	Stage 3	>180/120 mmHg	0.93	1.27	0.74	1.20	1.02	0.00
**TC/HDL**	Healthy	<5.0(M),	73.45	75.00	72.48	61.73	83.33	
		<4.5 (F)						
	Risk	>5.0 (M),	26.55	25.00	27.52	38.27	16.67	
		>4.5 (F)						
**LDL/HDL**	Healthy	<3.5 (M),	75.14	78.13	82.66	64.20	84.38	
		<3.0 (F)						
	Risk	>3.5 (M),	24.86	21.88	17.34	35.80	15.63	
		>3.0 (F)						
**Smoking**	Non-smoker		58.17	23.08	79.23	76.92	40.63	64.71
	Former		9.62	16.67	5.38	2.56	13.54	14.71
	Smoker		32.21	60.26	15.38	20.51	45.83	20.59
**FRS**	5-year risk ± 95% CI		4.35 ± 0.88	5.94 ± 1.69	3.37 ± 0.93	4.06 ± 1.17	4.59 ± 1.30	
**MetS**	Yes		44.63	36.76	49.54	33.33	54.17	
	No		55.37	63.24	50.46	66.67	45.83	

Approximately a third of the OA (35.98%) exhibited normal blood pressure, while 41.12% were pre-hypertensive, and 21.49% were hypertensive. Lipoprotein ratios TC/HDL and LDL/HDL, both used as predictors for cardiometabolic health, revealed 26.55% and 24.86% of OA were at high risk for poorer health. Framingham Risk Score (FRS) calculator that was recalibrated for an indigenous Australian cohort was utilized to determine the risk of OA developing cardiovascular risk over the next five years. The overall FRS for OA was 4.35% ± CI 0.88, with a slightly higher risk among OA men (5.94% ± CI 1.69) than women (3.37% ± CI 0.93). Metabolic syndrome (MetS) was diagnosed in 44.63% of the OA, of which women (49.54%) had a slightly higher prevalence compared to men (49.54%). Lipid profiles were not available for the urban Temuan due to fieldwork limitations.

### 3.2 Oral Microbiota

We analysed the V4 region of the 16S rRNA gene from 210 saliva samples (4 samples failed the QC threshold with <20,000 reads per sample). At 90% sample prevalence and 0.1% relative abundance detection threshold, 15 genera were identified as core constituents of the OA oral microbiota. Common oral bacteria such as *Streptococcus, Veillonella, Haemophilus, Prevotella, Gemella, Neisseria, Lactobacillales, Actinomyces* and *Fusobacterium* were identified (**Supplementary Figure 1**). Notably, three ‘uncultured bacterium’ were detected.

#### 3.2.1 Unexpectedly Low Alpha Diversity in OA Oral Microbiota

**Figure 1:**
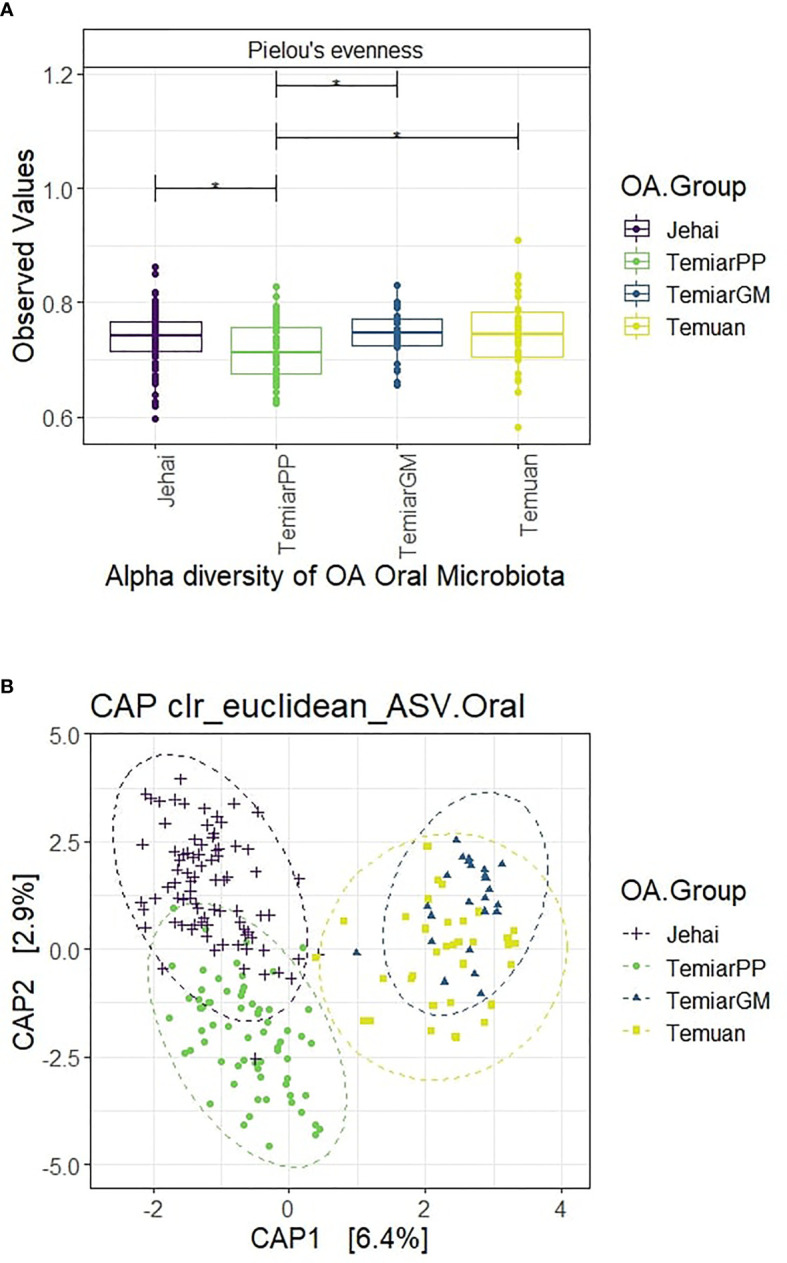
**(A)** Alpha diversity of OA oral microbiota unexpectedly low and homogenous (* p-value < 0.05). **(B)** Supervised CAP ordination plot shows distinct clusters with rural Jehai and Temiar PP microbiota grouping away from urban-like Temuan and Temiar GM.

Alpha diversity was measured using Pielou’s evenness, which combines microbial richness and evenness (**Figure 1A**). Semi-nomadic Jehai who lived in the rainforest had unexpectedly low oral microbiota comparable to sub-urban Temiar GM and urban Temuan. Rural Temiar PP, who lived near palm oil plantations, had the lowest alpha diversity compared to all OA communities (p<0.05).

#### 3.2.2 Rural OA Oral Microbiota Enriched in Uncharacterised Bacteria

Beta diversity was measured using clr-transformed Euclidean distance at the ASV level and visualised using a supervised CAP (Canonical analysis of principal coordinates) plot (**Figure 1B**). OA oral microbiota formed four distinct clusters with some overlap between semi-nomadic Jehai and Temiar PP. Surprisingly, the oral microbiota of sub-urban Temiar GM largely overlapped with urban Temuan despite differences in lifestyles and geographical locations. Rural Temiar PP oral microbiota formed a distinct cluster away from sub-urban Temiar GM without any overlap and clustered more closely with the semi-nomadic Jehai.

PERMANOVA multivariate test revealed that the centroids of the four OA groups were significantly different even after controlling for BMI and age groups (PERMANOVA Pseudo-F = 8.1576, R2 = 0.104, p-value =0.001). PERMDISP was used to test for homogeneity of sample dispersion and revealed significant within-group variation (PERMDISP F-stats = 8.15, p-value =0.003). This result is in line with the CAP ordination plot, where sample dispersions were visibly different. A further univariate differential abundance test using ALDEX2 was conducted to investigate bacteria genus differentially abundant between the OA groups.

Jehai microbiota were more similar to rural Temiar PP as no bacteria were significantly different, while sub-urban Temiar GM microbiota were more similar to urban Temuan. Urban-like microbiota Temuan and Temiar GM were enriched in bacteria genera *Corynebacterium, Prevotella* and *Mogibacterium* compared to rural-like microbiota Jehai and Temiar PP (BH-corrected p-value <0.01, effect size >1). On the other hand, the rural-like microbiota Temiar PP and Jehai were enriched in relatively novel and uncharacterised bacteria including an uncultured Porphyromonadaceae, Prevotella 2 and Candidatus Saccharibacteria bacterium UB2523 (BH-corrected p-value <0.01, effect size < -1).

### 3.3 OA Gut Microbiota

Targeted 16S rRNA sequencing of the V4 region was used to characterise 165 OA gut microbiota. Two samples failed to pass the QC threshold. Bacteria shared by at least 90% of the samples at 0.1% detection threshold revealed ten core genera. Notably, nine out of the ten core genera identified in at least 90% of the samples were from the phylum Firmicutes, class Clostridia. *Collinsella, Dorea, Blautia, Faecalibacterium, Lachnospiraceae*, *Ruminococcaceae* and *Subdoligranulum* were identified as the OA core gut constituent (**Supplementary Figure 2**).

#### 3.3.1 Semi-nomadic Jehai had the Highest Alpha Diversity Among the OA Gut Microbiota

**Figure 2:**
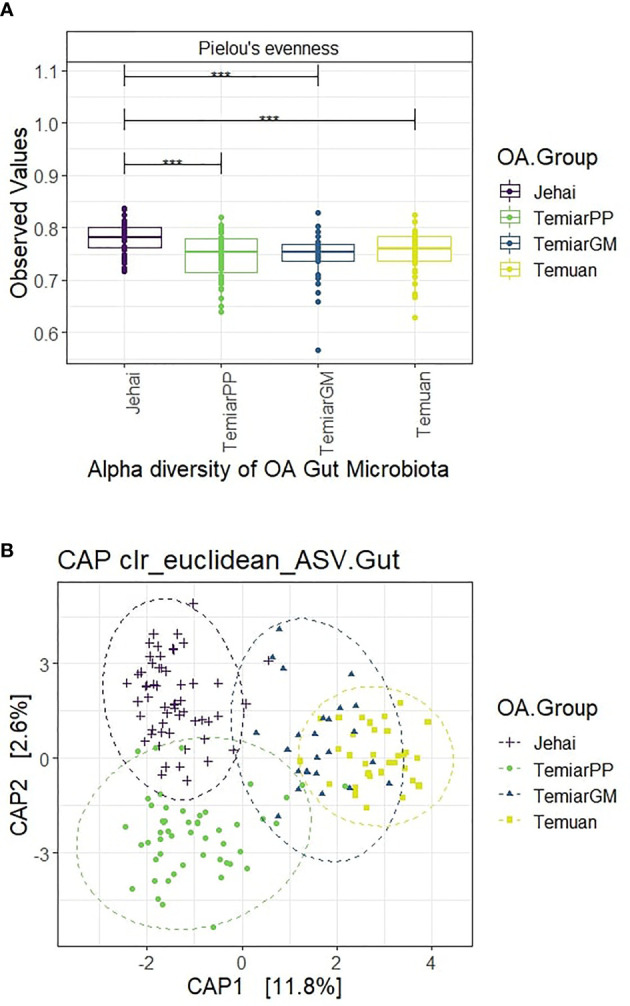
**(A)** Semi-nomadic Jehai had the highest alpha diversity, while urban Temuan had the lowest (*** p-value < 0.001). **(B)** OA gut microbiota formed four distinct clusters with some overlaps.

Semi-nomadic Jehai exhibited the highest alpha diversity among the four OA communities using Pielou’s evennesss (p<0.001). OA communities who lived nearer to urban areas had observably decreased alpha diversity, with urban Temuan exhibiting the lowest diversity (**Figure 2A**). Rural Temiar PP and sub-urban Temiar GM had similar alpha diversity.

#### 3.3.2 Rural OA Gut Microbiota Were Distinct From Urban-Like OA Microbiota

A supervised CAP ordination plot showed that the OA gut microbiota separated into four distinct clusters with overlaps (**Figure 2B**). The sub-urban Temiar GM appeared to have a mixture of ‘microbiota types’. Most of the Temiar GM gut microbiota overlapped with the urban Temuan’s except for a few individuals who appeared to have a more rural, Jehai-like microbiota. There was little overlap between the semi-nomadic Jehai and rural Temiar PP gut microbiota. Rural Temiar PP and sub-urban Temiar GM gut microbiota were rather distinct from each other.

The centroids of the OA groups were significantly different after controlling for BMI and age-group (PERMANOVA Pseudo-F = 8.7755, R2 = 0.141, p-value = 0.001). PERMDISP was used to test for homogeneity of sample dispersion and revealed significant within-group variation (PERMDISP F-stats = 16.476, p-value = 0.001).

Rural Jehai and Temiar PP microbiota were enriched in *Prevotella 2, Prevotella 9* and Lachnospiraceae ND3007 group when compared to urban-like microbiota of Temuan and Temiar GM. Jehai microbiota were enriched in an uncultured Porphyromonadaceae bacterium which was depleted in urban Temuan. Rural microbiota were also found mostly enriched in *Solobacterium*, with the exception when comparing Temiar PP against Temuan. Jehai gut microbiota were not significantly different from rural Temiar PP.

On the other hand, urban Temuan microbiota were enriched in several genera, including *Odoribacter, Blautia, Parabacteroides, Bacteroides* and Ruminococcaceae UCG-013 that were depleted in rural Jehai and Temiar PP microbiota. Sub-urban Temiar GM microbiota were enriched in *Parabacteroides* and *Bacteroides* compared to Jehai. Temiar GM microbiota were elevated in *Coprococcus 3* compared to Temiar PP microbiota.

There were no appreciable taxa differences in gut microbiota when samples were stratified by age-groups, smoking habit, or gender. However, overweight individuals had a higher abundance of two *Prevotella* genera, *Prevotella 9* and *Prevotella 2*, than obese individuals.

### 3.4 Sequencing Data Comparability

Three stool and three saliva samples were sequenced on V4 and V3-V4 regions (Gut n=6, Saliva n=6). Samples sequenced on the V3-V4 16S rRNA region were trimmed to the V4 region and compared. Stool appeared more susceptible to different extraction methods, 16S rRNA hypervariable region and sequencing platform as there were some minor differences observed in the trimmed sequences and their V4 counterparts at the Class level (**Supplementary Figure 3**). Saliva samples had more similar profiles despite differences in extraction method, 16S rRNA hypervariable region and sequencing platform. ALDEX2 revealed no significantly different bacteria genus between V4 and trimmed V4 reads. The ordination plot also revealed that the samples resembled each other (**Supplementary Figure 4**). Therefore, it does appear that despite differences in sample extraction and sequencing, V4 sequences and V3-V4 reads that were trimmed to the V4 region had microbiota compositions that were quite comparable.

### 3.5 Microbiota Controls

Two mock communities (ATCC MSA-1002) underwent the same PCR amplification procedure at the V3-V4 region as all the samples sequenced in 2017 and 2020, respectively. The negative controls were PCR blank, saliva kit blank and stool kit blank, all containing ultrapure water. The saliva and stool kit blanks underwent the same DNA extraction process as the saliva and stool samples.

Despite the same mock community being amplified and sequenced with the same protocol, there appears to be a batch effect. The top five genera found in the kit and PCR negative controls were *Pseudomonas* (50.96%), *Streptococcus* (17.57%), *Neisseria* (14.70%), *Prevotella 9* (10.21%) and *Prevotella 7* (9.67%). Full list of contaminants identified can be found in the **Supplementary File (Data Sheet 1)**.

To determine if the bacteria genus that tested differentially abundant in ALDEX2 reflected actual biological differences instead of contaminants, ALDEX2 results were cross-checked against the list of contaminant bacteria found in the negative controls. **Table 2** summarises the differentially abundant bacteria identified in the negative controls and the extent of the contamination. All contaminant genera had a relative abundance of less than 2%, except for *Blautia* and *Prevotella 9* found in stool-negative controls with a relative abundance of 2% and 6.4%, respectively.

**Table 2:** Summary of bacteria genera of differentially abundant bacteria genera which appeared significant in ALDEX2 analysis and appeared in the negative controls.

Saliva	Genus	Sample ID	Sample Type	Body Site	Abundance (%)
OTU_ID					
1363	*Campylobacter*	Saliva-neg	PCR	NA	0.1582
1967	*Corynebacterium*	Saliva-neg	Kit	Oral	0.0333
1624	*Actinomyces*	Saliva-neg	Kit	Oral	0.8975
565	*Prevotella*	Saliva-neg	Kit	Oral	1.4640
1137	*Peptostreptococcus*	Saliva-neg	Kit	Oral	0.0562
1655	*Alloprevotella*	Saliva-neg	Kit	Oral	1.4120
1662	*Alloprevotella*	LF-Negative	Kit	Oral	1.0638
342	uncultured Porphyromonadaceae bacterium	Saliva-neg	Kit	Oral	0.4596
2145	*Prevotella 2*	Saliva-neg	Kit	Oral	1.6717
**Gut**					
295	*Blautia*	Stool-neg	PCR	NA	2.0000
1913	*Prevotella 9*	Stool-neg	Kit	Gut	6.4384
2206	*Prevotella 2*	Stool-neg	Kit	Gut	1.0461
199	Lachnospiraceae ND3007 group	Stool-neg	Kit	Gut	0.0670
993	*Solobacterium*	Stool-neg	Kit	Gut	0.0593
350	uncultured Porphyromonadaceae bacterium	Stool-neg	Kit	Gut	0.0848

## 4 Discussion

Most OA communities live in relatively isolated villages that are only accessible by dirt roads. Hence, they have limited healthcare access. This may account for the scarcity of up-to-date health information about the OA communities. The information that is available may either be outdated or originate from a few OA villages that are more often visited by healthcare workers. The human microbiome is deeply entwined with host health. There have been many reports of cardiometabolic links to the microbiome in various urban cohorts (Hansen et al., 2015; Fei et al., 2019; Papadopoulou et al., 2020; Wang et al., 2021). Therefore it was important for us to ascertain if this may also apply to rural and indigenous populations such as the OA in Southeast Asia.

This current study reflected many previous reports where urbanised OA communities suffered from poorer cardiometabolic health. The prevalence of metabolic syndrome (MetS) among the OA of this study from 2017-2019 was approximately 44.63%, with a slightly higher prevalence among women than men (49.54% vs 36.76%). A more extensive study reported that from 2010-2016, MetS prevalence was approximately 40% among the urbanised OA (Aghakhanian et al., 2019). The MetS prevalence of this current study is not directly comparable to the larger study reported by Aghakhanian et al. (2019) due to the vast difference in sample size (629 vs 215). Yet, both studies demonstrate that MetS is increasingly prevalent among the OA, especially those living near towns and cities.

The Negrito hunter-gatherers, such as Jehai and Batek, were reportedly leaner but had a higher incidence of dyslipidaemia (Aghakhanian et al., 2019). Our more in-depth study reflected similar findings where Jehai had the lowest prevalence of general obesity but had twice the prevalence of dyslipidaemia and apparent risk for cardiovascular diseases compared to Temiar. However, studies suggest that Negritos, of which Jehai is a sub-group, may present with increased coronary risk and inflammatory biomarkers, yet had low pro-thrombosis and endothelial activation (Mokhtar et al., 2016; Mokhsin et al., 2018). These studies suggested that genetic factors may have resulted in lower HDL levels among Negritos, which was also observed in this current study. In contrast to settled, urban populations which comprise the majority of citizens in Malaysia, it remains a great challenge to set up longitudinal health studies to track smaller, semi-urbanised and more mobile communities.

The OA oral microbiota overall had unexpectedly low alpha diversity. The semi-nomadic Jehai had comparably low diversity, similar to urban Temuan and sub-urban Temiar GM. Intriguingly, Temiar PP scored the lowest in alpha diversity. Long term betel quid chewing has been associated with lower alpha diversity (Hernandez et al., 2017). The Temiar PP community reported that they chewed betel quid (Yeo et al., 2019). The Temuan and Jehai are also known to chew betel quid (fieldwork observation). Low alpha diversity has also been reported among rural oral microbiota from Sierra Leone and DRC Congo inhabitants, whereas higher alpha diversity was reported among Germans and Batwa pygmies (Li et al., 2014). Access to a wider variety of food sources in urban Germany and higher microbe exchange frequency among more dense populations were suggested as possible explanations (Li et al., 2014). It may seem that the combination of betel quid chewing, less diverse food source and low-density population may have resulted in a more homogenous microbiota and lower alpha diversity in the OA oral microbiota.

*Corynebacterium* and *Prevotella* were found elevated in urban-like oral microbiota of Temuan and Temiar GM. These are oral commensals that were identified in saliva negative controls, albeit at low relative abundance (<1.5%). *Mogibacterium* is a strict anaerobe found increased in urban-like OA microbiota and was absent in the negative controls. *Mogibacterium* has been associated with periodontitis and caries (Chen et al., 2018; Chen et al., 2020). It is premature to draw any conclusions because there are various species under each of these genera of which short 16S rRNA reads cannot distinguish. However, oral bacteria associated with oral diseases were found elevated in urban-like OA oral microbiota which may suggest that the more urbanised OA oral microbiota are at dysbiosis. Further studies may be warranted to investigate oral health among rural and urban OA communities.

It was interesting to note that rural microbiota of Temiar PP and Jehai were found enriched in relatively novel and uncharacterised bacteria such as uncultured Porphyromonadaceae, *Prevotella 2*, and Candidatus Saccharibacteria UB2523. These bacteria were not identified in the oral negative controls. Three ‘uncultured’ bacteria were also detected as core constituents of the OA oral microbiota. These findings may address gaps in oral microbiota knowledge which has usually focused on culturable bacteria. With oral microbiomes receiving much less attention than gut microbiomes, hence we know very little about the oral microbiomes of indigenous people (Nath et al., 2021). Increasing oral microbiome research among under-represented populations is necessary. These efforts may lead to improved oral health among people who suffer from a higher burden of oral diseases and provide new insights into novel bacteria species and mechanisms in oral bacteria-host interactions (Nath et al., 2021).

The OA gut microbiota provided further insights. Semi-nomadic Jehai had the highest alpha diversity, while the urban Temuan exhibited the lowest. This was in agreement with previous reports where high alpha diversity were documented among other indigenous populations such as Tanzania’s Hadza hunter-gatherers (Hansen et al., 2019), Nepal’s Chepang foragers (Jha et al., 2018), traditional Matsés hunter-gatherers and Tunapuco’s rural agriculturist population in Peru (Obregon-Tito et al., 2015). The intermediary microbiota observed in Temiar PP and Temiar GM communities were reported in Nepal’s traditional, semi-urbanised agriculturist population (Jha et al., 2018). Increasingly higher alpha diversity among gut microbiota of more rural populations was quite different from the trend observed in the OA oral microbiota.

Rural OA gut microbiota diverged from urban-like microbiota. Rural OA gut microbiota were enriched in *Prevotella 2, Prevotella 9* and Lachnospiraceae ND3007. Jehai gut microbiota had elevated levels of an uncultured Porphyromonadaceae bacterium, which were depleted in urban Temuan. *Solobacterium* was found in higher relative abundance in rural OA gut microbiota. *Solobacterium* is a recently described opportunistic anaerobic pathogen isolated from human faeces and an oral commensal that is very difficult to culture (Barrak et al., 2020). Not much is known about *Solobacterium* and its’ role in human health and disease except that it has been reported in a few cases of halitosis, bacteraemia and surgical wound infections (Barrak et al., 2020).

*Blautia* was found depleted in rural Jehai and Temiar PP gut microbiota but enriched in urban Temuan. It was also reported in low abundance among the Hadza hunter-gatherers compared to urban Italians (Schnorr et al., 2014). *Blautia* and *Bacteroides* were reportedly increased in association with the degree of urbanisation (Yong et al., 2021), which appears in line with our findings. However, some studies have found *Blautia* inversely associated with visceral fat (Ozato et al., 2019) and obesity (Benítez-Páez et al., 2020). This was not the case in our investigation where *Blautia* was depleted among the Jehai who were the leanest and had the lowest prevalence of obesity. There may be an ostensible association between *Blautia* and health (Liu et al., 2021). However, conflicting findings suggest that future investigations of *Blautia* would need to be advanced to the species or strain-level before associations are made.

*Bacteroides* and their close relatives *Parabacteroides* are major constituents of the human microbiota and were found to be elevated in urban Temuan gut microbiota. *Bacteroides* is predominantly found in the gastrointestinal tract, especially among people with ‘Western’ diets that are enriched in animal fat and protein (Wu et al., 2011). *Bacteroides* were also reported in lower abundance in the gut microbiomes of Hadza hunter-gatherers (Schnorr et al., 2014). Another study reported that a dietary-fibre deprived gut had more *Bacteroides* (Desai et al., 2016). These findings are in line with our findings as rural Temiar PP and Jehai consumed a lot of plant-based food, including tapioca, a variety of ferns that grow by the forest and other edible shoots.

The limitations of next-generation sequencing have been extolled by earlier reports, including batch effects, contamination from commercial and in-house extraction kits as well as the impact of sequencing region and platform (Edmonds and Williams, 2017; Bukin et al., 2019; Hornung et al., 2019). As there is no current consensus on how best to combine microbiome data produced by different methods for robust analysis, we included a small control to test for compatibility. Our results do show that gut and saliva samples that were sequenced originally on the V3-V4 region and trimmed to only the V4 region had comparable microbial compositions to sequenced samples on the V4 region. Saliva samples seemed to be less affected than stool samples, as previously reported (Lim et al., 2017; Rosenbaum et al., 2019; Videnska et al., 2019). The negative controls did reveal some contaminants, although at relatively low abundance. Despite best practices and extreme care taken during sample collection and DNA extraction, it may be unavoidable to keep the samples completely free from contaminants, especially when working in the field. Nonetheless, contaminants that appeared in the negative controls were marked explicitly in the results section differential abundance analysis. Describing and acknowledging the presence of contaminants (Hornung et al., 2019) in lieu of manually removing them *in silico* may be more appropriate and would minimise manipulation of the dataset.

The difference in sample size for oral and gut samples reflected the reality of fieldwork where saliva samples were evidently easier to collect than stool. Time and effort were required to establish a friendly and trusting relationship with the participants before they were comfortable with us. There was no association found between the OA microbiota and their cardiometabolic health or the presence of MetS due to the limited sample size. Nonetheless, it was necessary to lay some groundwork regarding the OA’s latest health trend and characterise their microbiomes. Future work may build on our study for more in-depth studies on the unique lifestyles and diets of the OA and the effects on their health and microbiomes.

## 5 Conclusion

Our findings aligned with previous studies where the OA are undergoing an epidemiological shift, especially among the urbanised OA who seem to suffer from poorer cardiometabolic health. MetS was prevalent among 44.63% of the OA. Jehai were reportedly leaner but had a higher incidence of dyslipidaemia. T2D was generally low among the OA at 6.9% while insulin resistance was estimated to be affecting 25.4% of participants. Lipoprotein ratios TC/HDL and LDL/HDL, which predicted cardiometabolic risk had a prevalence of 26.55% and 24.86% among the OA. FRS score estimated that the OA had a 4.35% ± CI 0.88 risk of developing cardiovascular diseases over the next five years, with a higher incidence among men. The OA oral microbiota were surprisingly homogenous with unexpectedly low alpha diversity among all four communities. The rural oral microbiota of Temiar PP and Jehai were mainly enriched in uncharacterised bacteria, including an uncultured Porphyromonadaceae bacterium, *Prevotella 2*, and Candidatus Saccharibacteria UB2523. The urban Temuan oral microbiota were elevated in potentially opportunistic oral bacteria such as *Corynebacterium, Prevotella*, and *Mogibacterium*, suggesting possible oral dysbiosis. The semi-nomadic Jehai gut microbiota revealed the highest alpha diversity, while urban Temuan exhibited the lowest. Rural OA gut microbiota were distinct from urban-like microbiota and were elevated in bacteria genera such as *Prevotella 2, Prevotella 9*, Lachnospiraceae ND3007, and *Solobacterium.* Urban Temuan microbiota were enriched in *Odoribacter, Blautia, Parabacetroides, Bacteroides* and Ruminococcacecae UCG-013.

## Data Availability Statement

Amplicon sequences used in this study have been uploaded to SRA under the BioProject number PRJNA778070. Metadata is available at https://bridges.monash.edu/articles/dataset/The_Oral_Gut_Microbiota_and_Cardiometabolic_Health_of_indigenous_Orang_Asli_communities/16959583

## Ethics Statement

The studies involving human participants were reviewed and approved by the Ministry of Health Malaysia (MOH) under the National Medical Research Registry (MNDR ID #09-23-3913), JAKOA (Department of Orang Asli Development) and Monash University Human Research Ethics Committee (MUHREC ID # 11794).

## Author Contributions

MP, YL contributed to study design and conception. MP and UP secured funds to support the study. BK, L-FY, SCL, MP, YL were involved in fieldwork and sample collection. L-FY, SCL, QA, SYL conducted lab work. L-FY performed the analysis and drafted the manuscript. All authors contributed to manuscript revision, read, and approved the submitted version.

## Funding

This work was supported by a research grant awarded to MEP by MOSTI Malaysia and internal grants awarded to L-FY by Jeffrey Cheah School of Medicine & Health Sciences (JCSMHS), Tropical Medicine and Biology (TMB) Multidisciplinary Platform, Monash University Malaysia Genomics Facility and Global Asia 21 Monash University Malaysia.

## Conflict of Interest

The authors declare that the research was conducted without any commercial or financial relationships that could be construed as a potential conflict of interest.

## Publisher’s Note

All claims expressed in this article are solely those of the authors and do not necessarily represent those of their affiliated organizations, or those of the publisher, the editors and the reviewers. Any product that may be evaluated in this article, or claim that may be made by its manufacturer, is not guaranteed or endorsed by the publisher.
