# Supplementary material for: The Oral, Gut Microbiota and Cardiometabolic Health of Indigenous Orang Asli Communities
Source: Front Cell Infect Microbiol. 2022 Apr 22;12:812345. doi: 10.3389/fcimb.2022.812345 (PMC9074829; doi:10.3389/fcimb.2022.812345)
Supplement: Supplementary file 2 [file DataSheet_2.docx]

Supplementary Material


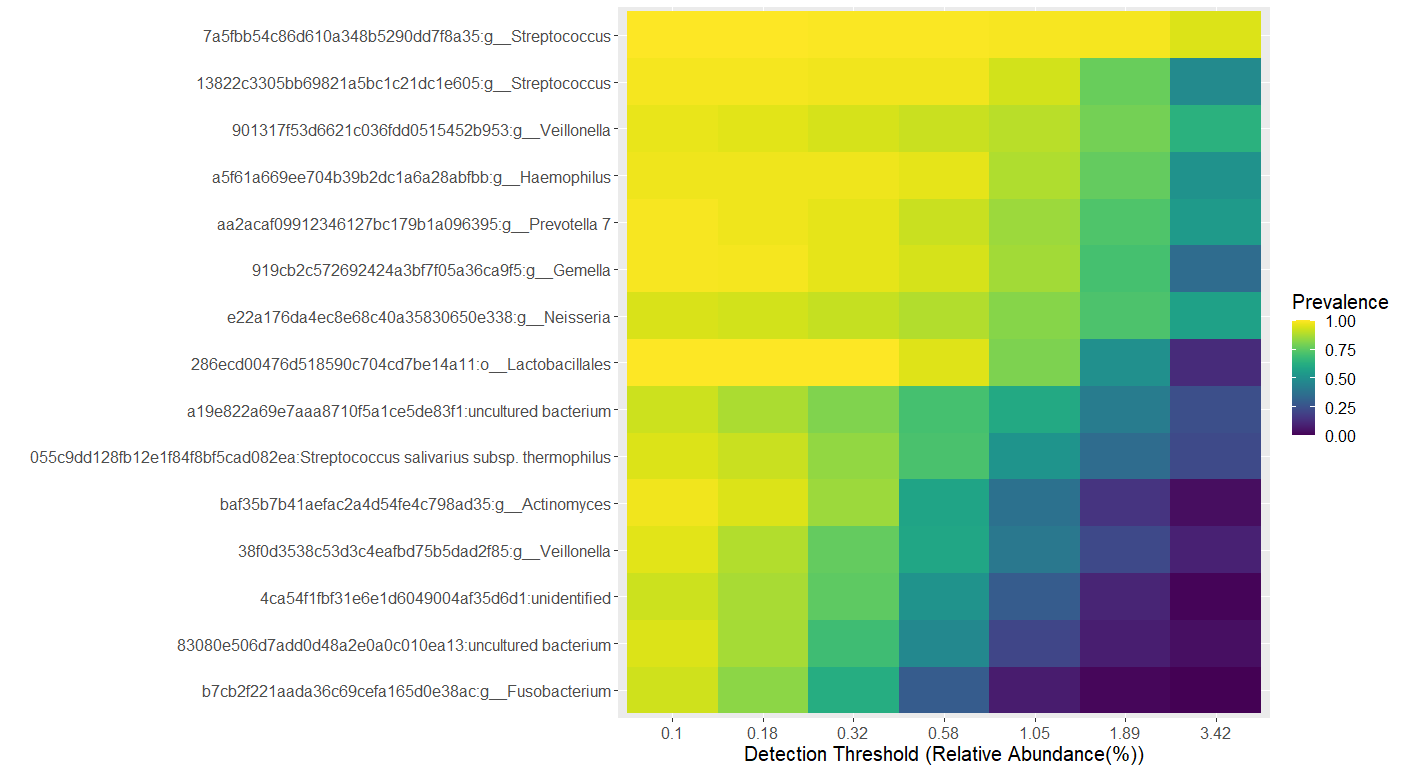


**Supplementary Figure 1.** Core oral microbiota constituents at the Genus level for at least 90% of the samples at 0.1% relative abundance detection threshold.


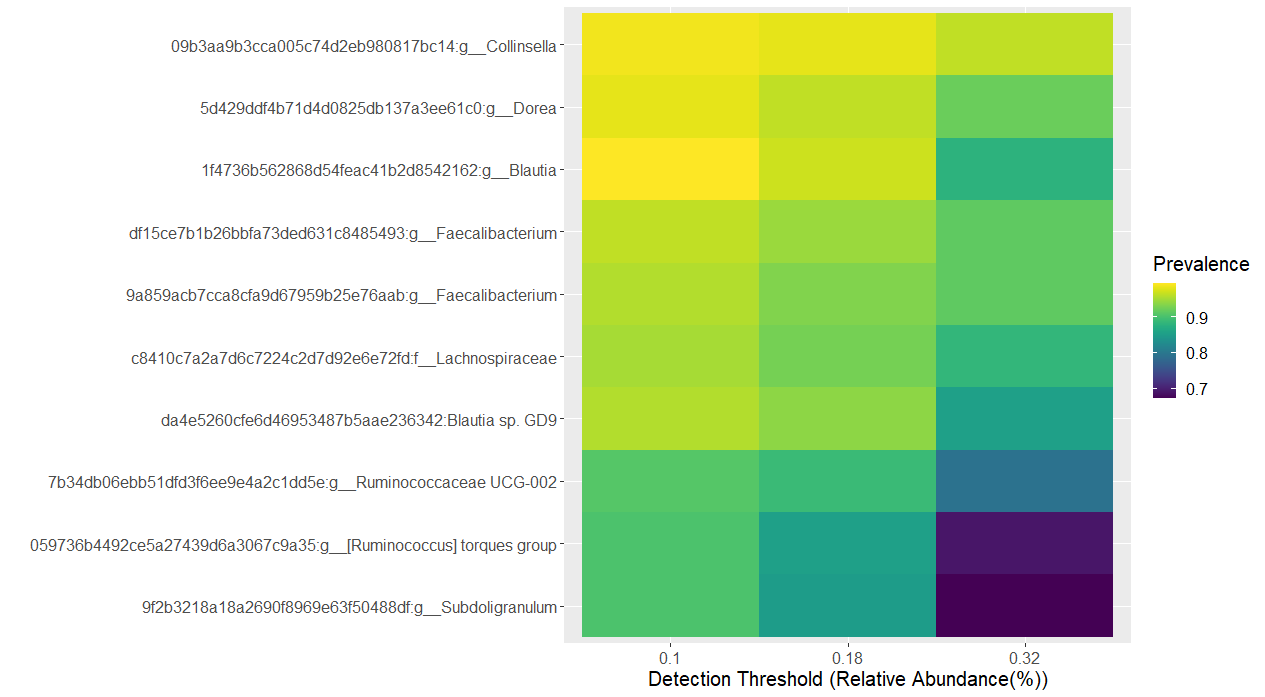


**Supplementary Figure 2.** Core gut microbiota constituents at the Genus level for at least 90% of the samples at 0.1% relative abundance detection threshold.

**Supplementary Table 1.** Pairwise comparison of OA communities reveal differentially abundant saliva bacteria genera using ALDEX2 (BH-corrected p-value <0.01 and effect size >1 or <-1).

|  | **Genus** | **Median clr of A^#^** | **Median clr of B^#^** | **effect** | **we.eBH** | **wi.eBH** |
| --- | --- | --- | --- | --- | --- | --- |
| **Jehai vs Temuan** | *Campylobacter** | 2.16763122 | 4.131676404 | 1.407222 | 1.77E-10 | 5.61E-13 |
|  | *Corynebacterium** | -0.500066048 | 2.653015978 | 1.286858 | 0.000525 | 3.63E-10 |
|  | *Actinomyces** | 2.778093294 | 5.645405564 | 1.105451 | 9.47E-10 | 2.54E-10 |
|  | *Prevotella** | 3.910704099 | 5.512669108 | 1.06383 | 5.24E-09 | 2.01E-10 |
|  | *Mogibacterium* | -5.93226897 | -0.618324232 | 1.010736 | 1.50E-06 | 3.47E-09 |
|  | Porphyromonadaceae uncultured* | -1.694333002 | -7.338729607 | -1.38314 | 3.24E-08 | 3.04E-11 |
|  | *Prevotella 2** | 0.33947362 | -7.294457009 | -2.08066 | 8.12E-11 | 8.15E-14 |
|  | Candidatus Saccharibacteria bacterium UB2523 | -2.273947217 | -7.326312213 | -1.16308 | 2.29E-07 | 1.64E-09 |
|  | Lentimicrobiaceae unidentified | -1.467537763 | -7.350531936 | -1.09245 | 2.74E-07 | 1.64E-08 |
| **Jehai vs TemiarGM** | *Corynebacterium** | -0.504361837 | 2.88986973 | 1.728029 | 0.001233 | 1.23E-09 |
|  | *Prevotella** | 3.911411123 | 5.512963911 | 1.385742 | 3.61E-09 | 7.06E-10 |
|  | *Peptostreptococcus** | 0.316691682 | 2.144257044 | 1.036108 | 3.60E-06 | 5.01E-07 |
|  | *Mogibacterium* | -5.922086449 | -0.302276587 | 1.6123 | 6.58E-20 | 2.23E-11 |
|  | Porphyromonadaceae uncultured* | -1.691482449 | -8.547363103 | -1.88637 | 1.47E-07 | 3.78E-11 |
|  | *Prevotella 2** | 0.342605791 | -7.813455872 | -2.35786 | 2.73E-08 | 1.75E-11 |
|  | Candidatus Saccharibacteria bacterium UB2523 | -2.277916993 | -8.54652133 | -1.61685 | 1.47E-07 | 2.65E-10 |
|  | SR1 (Absconditabacteria) (Phylum) | -1.493180901 | -8.564648551 | -1.20048 | 1.01E-06 | 3.32E-07 |
|  | Lentimicrobiaceae unidentified | -1.470012372 | -8.58279656 | -1.4566 | 2.63E-07 | 1.33E-08 |
| **Temiar vs Temuan** | *Campylobacter** | 2.139979433 | 4.138672914 | 1.314972 | 5.97E-12 | 1.20E-11 |
|  | *Corynebacterium** | -0.72693244 | 2.652346306 | 1.306579 | 0.000431 | 1.33E-09 |
|  | *Prevotella** | 3.350969384 | 5.50739596 | 1.268683 | 8.42E-12 | 6.87E-11 |
|  | *Mogibacterium* | -6.269334896 | -0.62160277 | 1.152272 | 3.07E-06 | 2.61E-09 |
|  | Porphyromonadaceae uncultured* | -1.10919176 | -7.325973728 | -1.56053 | 5.58E-09 | 1.59E-11 |
|  | *Prevotella 2** | 0.147821669 | -7.320420306 | -1.84287 | 1.88E-09 | 4.18E-12 |
|  | Candidatus Saccharibacteria bacterium UB2523 | -1.527025681 | -7.308159352 | -1.10692 | 4.13E-07 | 2.11E-08 |
| **Temiar vs TemiarGM** | *Corynebacterium** | -0.728038296 | 2.890748377 | 1.73868 | 0.000585 | 3.11E-09 |
|  | *Alloprevotella** | 2.238465264 | 4.835386013 | 1.448045 | 5.42E-13 | 2.86E-09 |
|  | *Prevotella** | 3.347604267 | 5.510315022 | 1.506326 | 4.28E-12 | 9.01E-10 |
|  | *Mogibacterium* | -6.275329923 | -0.291390612 | 1.844286 | 1.02E-18 | 3.41E-11 |
|  | Porphyromonadaceae uncultured* | -1.104591349 | -8.514786221 | -2.12502 | 2.35E-08 | 7.97E-11 |
|  | *Prevotella 2** | 0.148021047 | -7.787262937 | -2.03924 | 1.84E-08 | 2.13E-10 |
|  | Candidatus Saccharibacteria bacterium UB2523 | -1.527203395 | -8.510078529 | -1.5344 | 1.48E-07 | 6.36E-09 |

**A meaning the former of the pair, B being the later of the pair*

**Bacteria genera identified in negative controls at <1.5% relative abundance*

**Supplementary Table 2.** Pairwise comparison of OA communities reveal differentially abundant gut bacteria genera using ALDEX2 (BH-corrected p-value <0.01 and effect size >1 or <-1).

| Group | Genus | Median clr of A^#^ | Median clr of B^#^ | effect | we.eBH | wi.eBH |
| --- | --- | --- | --- | --- | --- | --- |
| Jehai vs Temuan | *Sutterella* | -0.882 | 5.690 | 1.101 | 5.86E-07 | 2.56E-08 |
|  | *Odoribacter* | -4.504 | 3.306 | 1.372 | 1.42E-08 | 6.29E-10 |
|  | *Blautia** | 5.807 | 7.723 | 1.047 | 1.20E-07 | 1.73E-09 |
|  | *Lachnoclostridium* | 1.300 | 4.531 | 1.070 | 0.001156 | 4.96E-08 |
|  | *Parabacteroides* | -2.827 | 5.941 | 1.759 | 2.11E-18 | 4.48E-13 |
|  | *Bacteroides* | 2.216 | 7.811 | 1.472 | 6.93E-11 | 5.03E-11 |
|  | Ruminococcaceae UCG-013 | -5.103 | 2.323 | 1.149 | 2.87E-07 | 2.83E-08 |
|  | *Prevotella 2** | 5.682 | -5.453 | -2.888 | 1.10E-13 | 4.75E-13 |
|  | *Prevotella 9** | 8.082 | -0.465 | -2.145 | 2.78E-11 | 4.73E-13 |
|  | Lachnospiraceae ND3007 group* | 1.038 | -5.423 | -1.601 | 6.97E-09 | 4.59E-11 |
|  | *Solobacterium** | 2.290 | -5.187 | -1.254 | 1.27E-07 | 5.50E-08 |
|  | uncultured Porphyromonadaceae bacterium* | 3.464 | -5.590 | -1.219 | 9.55E-09 | 2.12E-07 |
| Jehai vs TemiarGM | *Parabacteroides* | -2.771 | 2.942 | 1.113 | 1.22E-05 | 1.54E-07 |
|  | *Bacteroides* | 2.282 | 5.610 | 1.106 | 1.39E-06 | 1.68E-07 |
|  | Lachnospiraceae ND3007 group | 1.112 | -6.070 | -2.095 | 2.66E-07 | 1.40E-10 |
|  | *Prevotella 9** | 8.154 | 0.990 | -2.319 | 1.08E-07 | 1.58E-10 |
|  | *Prevotella 2** | 5.754 | -1.296 | -1.499 | 7.77E-07 | 9.44E-10 |
|  | *Solobacterium* | 2.354 | -4.849 | -1.309 | 1.15E-06 | 1.97E-07 |
| Temiar vs TemiarGM | *Coprococcus 3* | 1.544 | 4.104 | 1.065 | 1.87E-06 | 1.53E-06 |
|  | Lachnospiraceae ND3007 group* | 1.122 | -6.027 | -2.105 | 1.35E-07 | 3.42E-10 |
|  | *Prevotella 9** | 8.961 | 0.996 | -2.423 | 1.87E-08 | 3.20E-10 |
|  | *Prevotella 2** | 6.641 | -1.341 | -1.575 | 1.85E-07 | 1.27E-09 |
|  | *Solobacterium** | 1.030 | -4.877 | -1.008 | 4.93E-05 | 1.43E-05 |
| Temiar vs Temuan | *Odoribacter* | -3.780 | 3.375 | 1.162 | 8.87E-08 | 2.52E-08 |
|  | *Blautia** | 5.582 | 7.776 | 1.048 | 5.80E-08 | 1.12E-08 |
|  | *Parabacteroides* | -0.137 | 6.008 | 1.243 | 9.39E-12 | 1.85E-10 |
|  | *Bacteroides* | 3.350 | 7.864 | 1.060 | 7.17E-08 | 3.06E-08 |
|  | Ruminococcaceae UCG-013 | -5.166 | 2.394 | 1.215 | 3.69E-08 | 1.84E-08 |
|  | Lachnospiraceae ND3007 group* | 1.123 | -5.318 | -1.680 | 4.37E-09 | 1.75E-10 |
|  | *Prevotella 9** | 8.961 | -0.389 | -2.252 | 5.30E-12 | 2.91E-12 |
|  | *Prevotella 2** | 6.641 | -5.357 | -2.816 | 1.98E-14 | 4.50E-12 |
| Obese vs Overweight | *Prevotella 9** | 1.943 | 8.840 | 1.162 | 4.53E-11 | 2.52E-09 |
|  | *Prevotella 2** | -3.039 | 6.598 | 1.133 | 3.30E-10 | 3.24E-09 |

*^#^A is the former and B is the later in pairwise comparison*

**Bacteria genera were identified in negative controls. Prevotella 9 at 6.4%, the rest <2% relative abundance*

Quality control


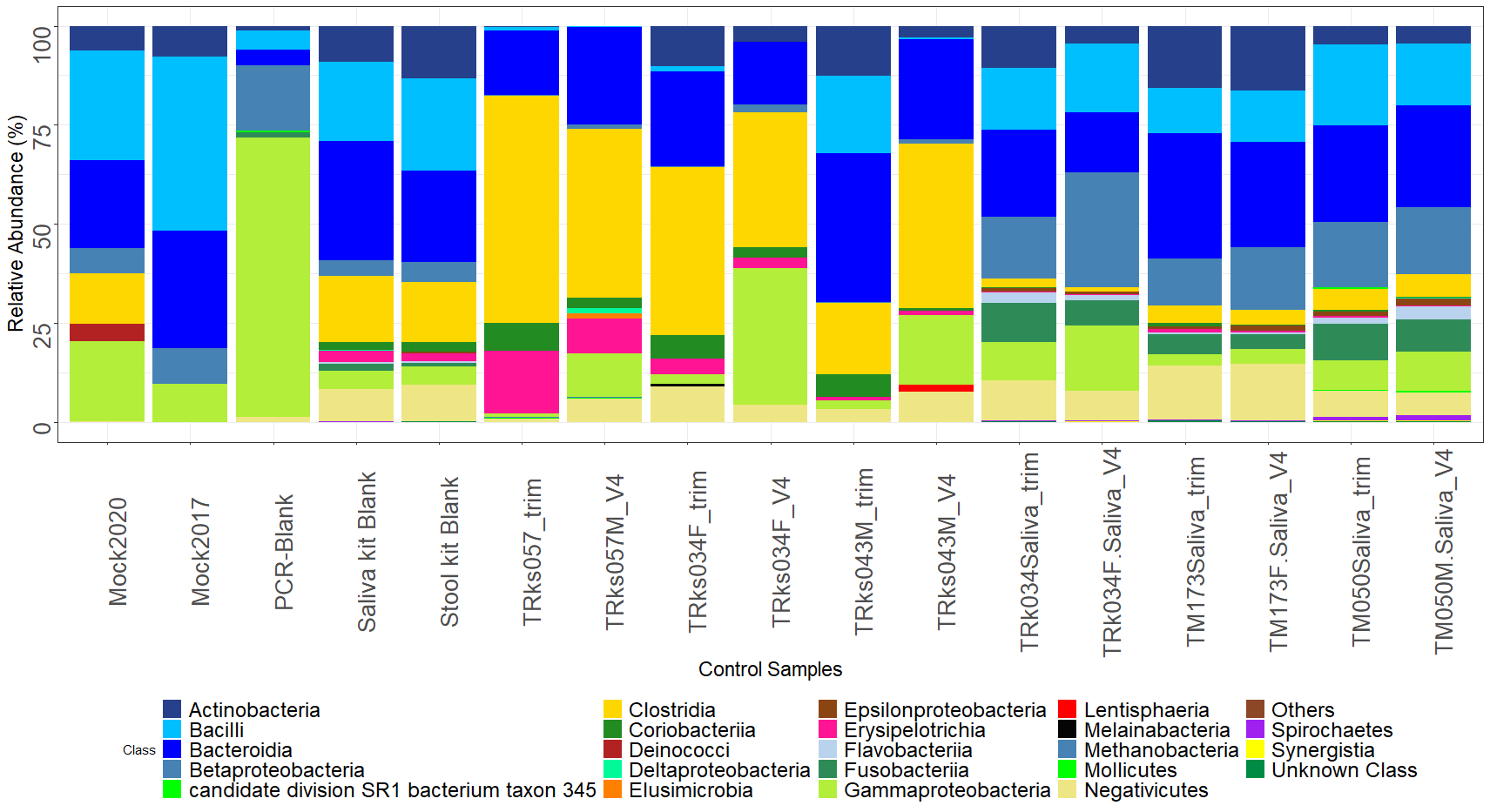


**Supplementary Figure 3.** Microbiota controls taxonomy bar plot


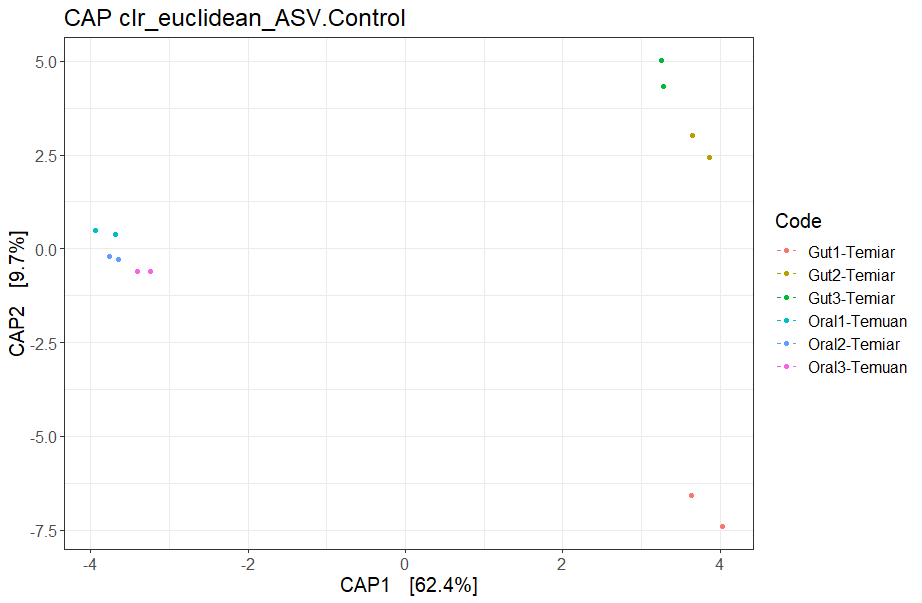


Oral

**Supplementary Figure 4.** Each colour pair indicates the same sample, one sequenced on V4 and another on V3-V4 trimmed to V4. Ordination plot shows that the same samples analysed on the V4 region still cluster together despite difference in extraction method and sequencing platform.
